# Peri-implant tissue conditions following transcrestal and lateral sinus floor elevation: 3-year results of a bi-center, randomized trial

**DOI:** 10.1007/s00784-021-04364-y

**Published:** 2022-01-10

**Authors:** Roberto Farina, Anna Simonelli, Giovanni Franceschetti, Luigi Minenna, Gian Pietro Schincaglia, Orio Riccardi, Leonardo Trombelli

**Affiliations:** 1 Operative Unit of Dentistry, Azienda Unità Sanitaria Locale (A.U.S.L.) of Ferrara, Ferrara, Italy; 2 Research Centre for the Study of Periodontal and Peri-Implant Diseases, University of Ferrara, Ferrara, Italy; 3 Department of Periodontology, Case Western Reserve University, OH Cleveland, USA; 4 Private Practice Torre Pedrera, Rimini, Italy

**Keywords:** Maxillary sinus, Surgical procedures, Dental implants, Bone regeneration, Peri-implantitis, Mucositis

## Abstract

**Objectives:**

The present study was performed to comparatively evaluate the peri-implant bone stability and conditions of marginal tissues at 3 years following transcrestal and lateral sinus floor elevation (tSFE and lSFE, respectively).

**Materials and methods:**

Patients included in a parallel-arm randomized trial comparatively evaluating tSFE and lSFE were recalled at 3 years post-surgery. Twenty-one and 24 patients in tSFE and lSFE groups, respectively, participated in the follow-up visit. Peri-implant bone support was evaluated as the proportion of the entire implant surface in direct contact with the radiopaque area (totCON%) on 3-year periapical radiographs. The conditions of the marginal peri-implant tissues at 3-year visit were classified as peri-implant health, peri-implant mucositis, or peri-implantitis.

**Results:**

At 3 years, both groups showed an implant survival rate of 100%. Median totCON% was stable at 3 years, being 100% in both groups (*p* = 0.124). Peri-implant health and mucositis were diagnosed in 10 (47.6%) and 11 (52.4%) patients, respectively, in the tSFE group, and in 8 (33.3%) and 16 (66.7%) subjects, respectively, in the lSFE group (*p* = 0.502).

**Conclusions:**

At 3 years following surgery, implants placed concomitantly with tSFE and lSFE fully maintain peri-implant bone support. Peri-implant mucositis was the most prevalent condition, with a similar prevalence between groups.

Clinical relevance.

Based on 3-year data on peri-implant bone support and prevalence of peri-implant diseases, the study suggests that tSFE and lSFE represent two equally valid options for the rehabilitation of the posterior maxilla. ClinicalTrials.gov ID: NCT02415946.

## Introduction

Maxillary sinus floor elevation with transcrestal and lateral access (tSFE and lSFE, respectively) are two validated options for vertical bone augmentation in the atrophic posterior maxilla. Both techniques were associated with high implant survival rates at long-term follow-up intervals [1, 2].

Radiographic analyses consistently indicate that, after its displacement with tSFE, the dimensions of the endo-sinusal grafted area tend to reduce overtime, although this tendency did not consistently reach statistical significance in all studies [3–8]. Also, it was shown that this reduction may lead to the exposure of the endo-sinusal portion of the implant [4, 6, 8]. Similar findings were reported for sites undergoing lSFE [9].

To date, the clinical outcomes of tSFE and lSFE have been compared in several trials [7, 10–21]. Among these, few randomized trials comparatively evaluated the impact of the dimensional reduction of the grafted area on peri-implant bone support at a follow-up of at least 2 years following tSFE and lSFE [14, 22]. In particular, endo-sinus bone–implant contact rates (as assessed radiographically) were similarly high and stable through the observation period, and amounted to 97.19% and 98.15% for tSFE and lSFE groups, respectively, at 2 years. Also, the 2-year position of the crestal bone level was found always coronal to the implant shoulder except for one patient in lSFE group [22]. Interestingly, a recent multivariate analysis conducted within a multicenter cross-sectional study identified the surgical access to the maxillary sinus as a factor influencing the risk for peri-implantitis occurrence [23]. In particular, lSFE was associated with significantly higher risk (OR = 6.75) for peri-implantitis compared to tSFE at a mean follow-up of 67.65 months from surgery. In the interpretation of their findings, the authors attributed increased peri-implantitis risk for lSFE to the combined effect of factors that may have determined early marginal bone loss: (i) extensive full thickness flap and a prolonged surgical time, (ii) compromised blood supply to the residual bone crest, and (iii) mechanical and thermal stress of the bone during underpreparation of the implant bed to obtain implant primary stability [23]. The evaluation of the incidence of peri-implant biological complications around implants undergoing tSFE vs lSFE within the context of a randomized trial remains confined to a limited number of studies [19, 22].

Recently, we performed a bi-center, parallel-arm, randomized trial comparatively evaluating tSFE and lSFE when applied concomitantly with implant placement at sites with limited (3–6 mm) residual bone. The results related to the clinical outcomes, morbidity, costs, and aspects of quality of life have already been published [15–17]. Based on the same cohort, the present study presents data related to (i) peri-implant bone stability and (ii) the conditions of the peri-implant marginal tissues at 3 years following tSFE and lSFE.

## Materials and methods

### Experimental design, ethical aspects, and trial registration

The present study consists of the 3-year evaluation of patients included in a bi-center, parallel-arm, single-blind, randomized trial comparatively evaluating tSFE and lSFE in terms of radiographic outcomes, intra- and postoperative morbidity, costs, and impact on specific aspects of quality of life [15–17]. The experimental protocol was approved by the Local Ethical Committees of Ferrara (protocol number: 140386) and Modena-Reggio Emilia, Italy (protocol number: 144/14). Each patient provided a written informed consent before participation. All the clinical procedures were performed in accordance with the standards of the Declaration of Helsinki, and the manuscript was prepared according to the CONSORT guidelines. The study was registered in www.clinicaltrials.gov (study ID: NCT02415946).

### Study population

Patients were consecutively treated at two University Hospitals (Ferrara and Modena, Italy) according to the selection criteria reported by Farina et al. [15]. Each patient contributed the study with one maxillary quadrant with ≥ 1 maxillary posterior site edentulous for at least 6 months and showing a residual bone height (RBH) of 3–6 mm.

### Clinical procedures

In patients assigned to receive tSFE, prosthetically guided preparation of the implant site/s was performed according to a validated technique based on a standardized sequence of instruments (*Smart Lift*; Meta CGM, Reggio Emilia, Italy) [8, 15–17, 24–32]. The tSFE procedure was combined with a plug of collagen matrix (Mucograft Seal®; Geistlich Pharma, AG, Wolhusen, Switzerland), which was placed in the future implant site before the fracture of the sinus floor, and an amount of deproteinized bovine bone mineral (DBBM; Bio-Oss® spongiosa granules, particle size 0.25–1.0 mm; Geistlich Pharma, AG, Wolhusen, Switzerland) which was pre-determined on the programmed extent of implant penetration into the sinus [15–17, 28]*.*

In patients assigned to receive lSFE, the antrostomy was used to place DBBM (Bio-Oss® spongiosa granules, particle size 0.25–1.0 mm or 1–2 mm; Geistlich Pharma, AG, Wolhusen, Switzerland) under the elevated sinus membrane. After prosthetically guided implant bed preparation according to the sequence of burs recommended by the implant manufacturer (Thommen Medical AG; Grenchen, Switzerland), the antrostomy was covered with a resorbable collagen membrane (Bio-Gide; Geistlich Pharma, AG, Wolhusen, Switzerland).

In both tSFE and lSFE groups, implants (SPI Inicell Element^©^; Thommen Medical AG, Grenchen, Switzerland) were inserted immediately after the completion of the grafting procedure. The healing protocol (submerged or transmucosal) was left at the operator’s discretion.

Implants placed with a submerged healing protocol at day 0 were surgically exposed at 20 weeks post-surgery, and a healing abutment was positioned.

Between week + 24 and week + 32 (6-month visit), implants were loaded with a provisional or definitive, cemented or screw-retained restoration (according to their treatment plan).

At 1-year visit, patients received personalized indications regarding their supportive periodontal therapy (SPT) program based on their periodontal risk level [33–35], and were left free to perform SPT at the center where they underwent surgery or other dental settings.

At 3 years, patients were recalled for a follow-up visit where clinical and radiographic assessments were performed to evaluate the conditions of peri-implant tissues.

### Radiographic and clinical measurements

Clinical and radiographic measurements were performed by a single, trained examiner (G.F.) who had previously undergone a calibration session on a sample of patients not included in the study and had participated as examiner in previous clinical trials including radiographic assessments of the outcomes of sinus lift procedures [8, 15, 16, 27–32].

#### Radiographic measurements

Periapical radiographs were taken at 1 and 3 years post-surgery with a paralleling technique using a Rinn film holder with a rigid film‐object X‐ray source. All available radiographs were scanned, digitized, stored at a resolution of 600 dpi, and analyzed using an image processing software (NIS Elements® v4.2; Nikon Instruments).

On each radiograph, the following measurements were performed:

- Peri-implant marginal bone level at the mesial (mMBL) and distal (dMBL) aspects of the implant: distance (in mm) from the apical margin of the implant shoulder to the first bone-to-implant contact at the mesial and distal aspect of the implant, respectively. To account for radiographic distortion, mMBL and dMBL were adjusted for a coefficient derived from the ratio: true length of the implant/radiographic implant length (rIL);
- Proportion of the entire implant surface in direct contact with the radiopaque area (totCON%): derived as the ratio (%) between the length (mm) of the implant surface in direct contact with the peri-implant radiopaque area (native bone + newly formed tissue) and the extent of implant surface [8].

#### ***Clinical ***measurements

At 3-year visit, the following clinical parameters were assessed using a manual periodontal probe (UNC15; Hu-Friedy, Chicago, USA) with a probing force of approximately 15 g at 6 sites (mesio-buccal, buccal, disto-buccal, disto-lingual, lingual, mesio-lingual) for each implant placed concomitantly with tSFE or lSFE:

- Probing depth (PD), measured in mm from the margin of the peri-implant mucosa to the bottom of the peri-implant sulcus/pocket;
- Bleeding on probing (BoP), recorded as positive (BoP +) when bleeding of the peri-implant mucosa was detected at the implant site after PD assessment;
- Suppuration on probing (SoP), recorded as positive (SoP +) when pus was detected at the implant site after PD assessment.

Mucosal recession (REC) was measured at the buccal aspect of the implant as the distance between the peri-implant mucosal margin and the implant-abutment junction (whenever visible), and recorded to the nearest mm.

Clinical measurements were assessed without removing the implant-supported prosthesis.

### Diagnosis related to the conditions of the peri-implant marginal tissues

Based on data on interproximal bone loss, PD, BoP, and SoP, the conditions of the peri-implant tissues at 3-year visit were retrospectively classified according to Berglundh et al. [36]: peri-implant health (i.e., no interproximal bone loss > 0.5 mm compared to 1-year radiograph, no increase in PD compared to 1-year visit, and no BoP + and/or SoP + sites); peri-implant mucositis (i.e., no interproximal bone loss > 0.5 mm compared to 1-year radiograph and at least 1 BoP + and/or SoP + site); or peri-implantitis (i.e., interproximal bone loss > 0.5 mm compared to 1-year radiograph, increased PD compared to 1-year visit, and at least 1 BoP + and/or SoP + site).

### Statistical analysis

Details of sample size calculation (which was based on a radiographic outcome measure) for the *intention-to-treat* (ITT) study population have been reported in a previous publication [16]. In the present study, a *per protocol* (PP) analysis was performed, including all patients undergoing the experimental protocol with no major deviations and attending the 3-year follow-up visit. The patient was regarded as the statistical unit. For patients receiving two implants concomitantly with sinus floor elevation in the experimental quadrant, only the implant which was previously selected for the 1-year follow-up study [16] was included for the 3-year evaluation. Since all numerical variables showed a non‐normal and non‐symmetric distribution, they were expressed as median and interquartile range (IR). For the primary outcome measure (totCON%), descriptive statistics incorporated also mean values.

totCON% was compared either within (i.e., 1-year vs 3-year) or between groups. Changes from 1-year to 3-year visit in mMBL and dMBL were calculated. mMBL and dMBL were compared either within or between groups. For the formulation of peri-implant diagnosis, the greatest change at either mMBL or dMBL was considered to determine if interproximal bone loss was > 0.5 mm or ≤ 0.5 mm. Within-group comparisons were performed by the Wilcoxon test, and treatment groups were compared using the *χ*^2^ test or Fisher’s exact test for categorical variables and the Mann–Whitney *U* test for numerical and ordinal variables. The level of statistical significance was fixed at 0.05.

## Results

### Study population

**Fig. 1:**
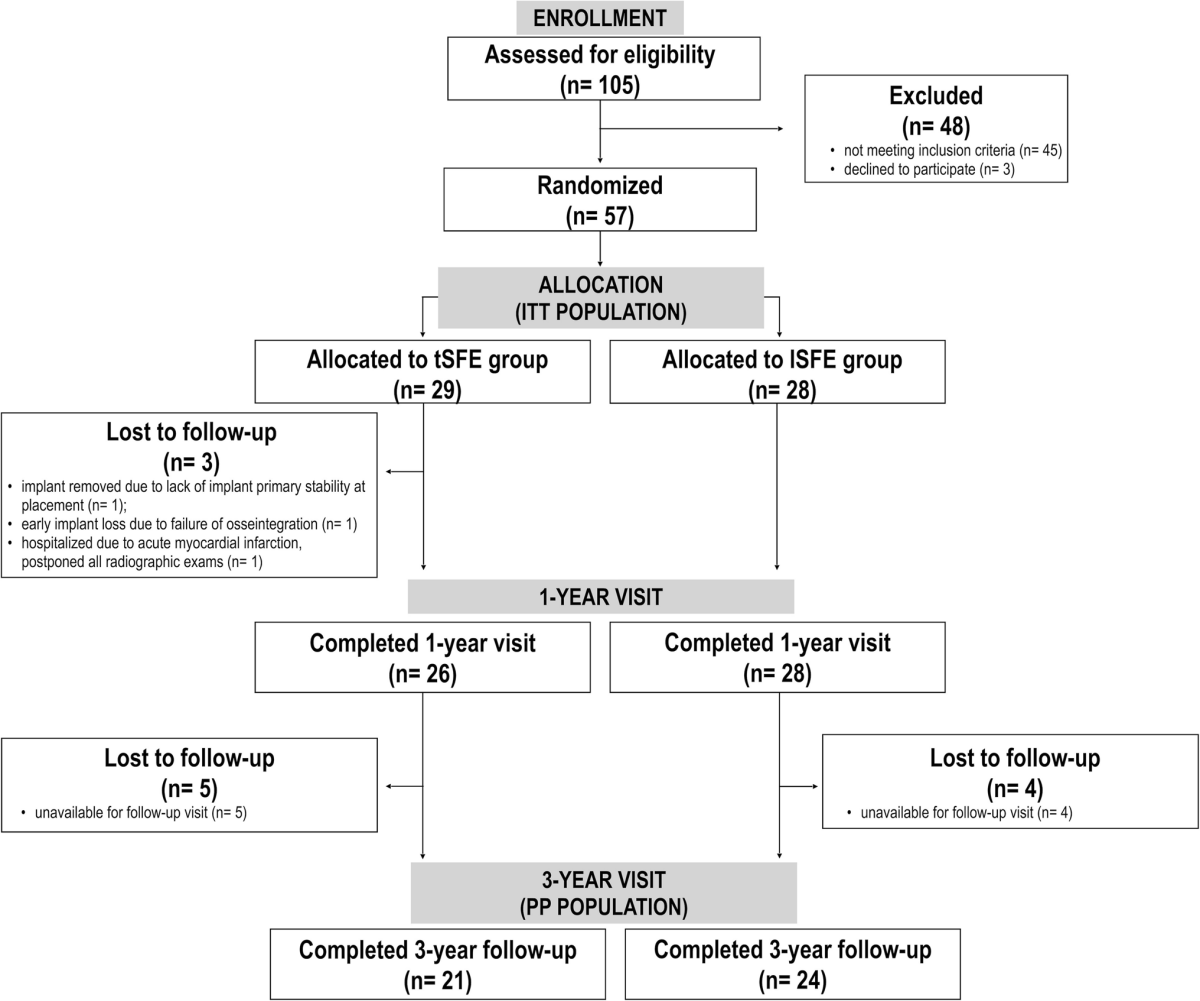
Flow chart of patient inclusion and follow-up

**Fig. 2:**
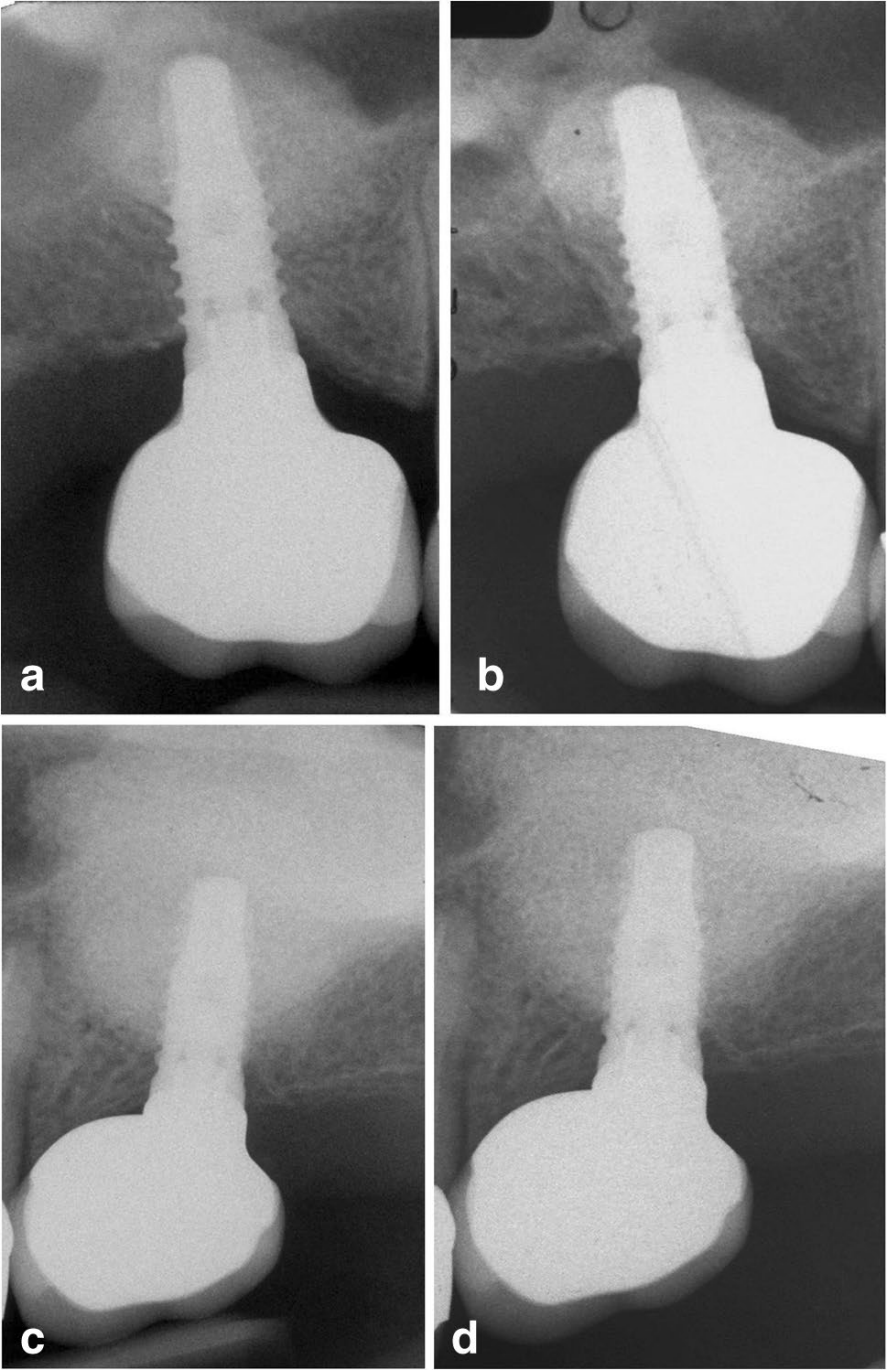
One- and 3-year follow-up periapical radiographs of two representative tSFE and lSFE cases. **a** tSFE, 1 year. **b** tSFE, 3 years. **c** lSFE, 1 year. **d** lSFE, 3 years

The PP study population consisted of 21 patients in the tSFE group and 24 patients in the lSFE group (Fig. 1). The patient- and implant-related characteristics of the PP population are reported in Table 1, and did not show significant differences between groups. The 1- and 3-year periapical radiographs of two paradigmatic tSFE and lSFE cases are illustrated in Fig. 2.

**Table 1:** Patient and implant characteristics of the *per protocol* study population. Values are expressed as median (IR) or frequencies

	tSFE group (*n* = 21)	lSFE group (*n* = 24)	*p* value
Age (years)	51.0 (49.0–60.0)	53.5 (49.8–62.5)	0.600
Gender (no. of males/females)	12/9	9/15	0.308
Smoking (no. of never smoked/former smokers/current smokers)	17/2/2	20/2/2	1
RBH (mm)	4.0 (3.9–5.1)	4.5 (4.0–5.4)	0.275
Implant length (mm)	9.5 (9.5–11.0)	9.5 (9.5–11.0)	0.431
Implant diameter (mm)	4.0 (4.0–4.0)	4.0 (4.0–4.0)	0.134

### Implant survival

The implant survival rate was 100% in both groups.

### totCON%

**Fig. 3:**
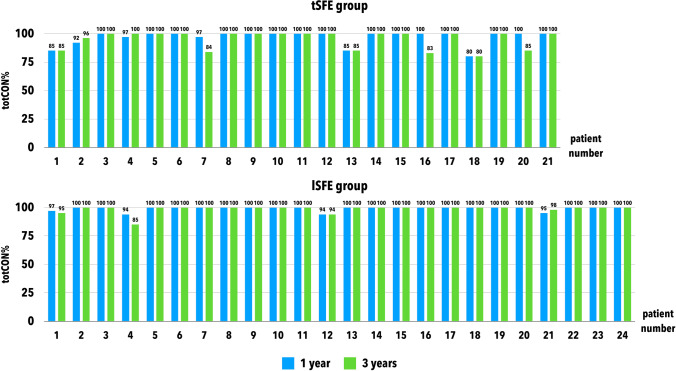
Raw data related to 1-year and 3-year totCON% values in tSFE and lSFE groups

**Fig. 4:**
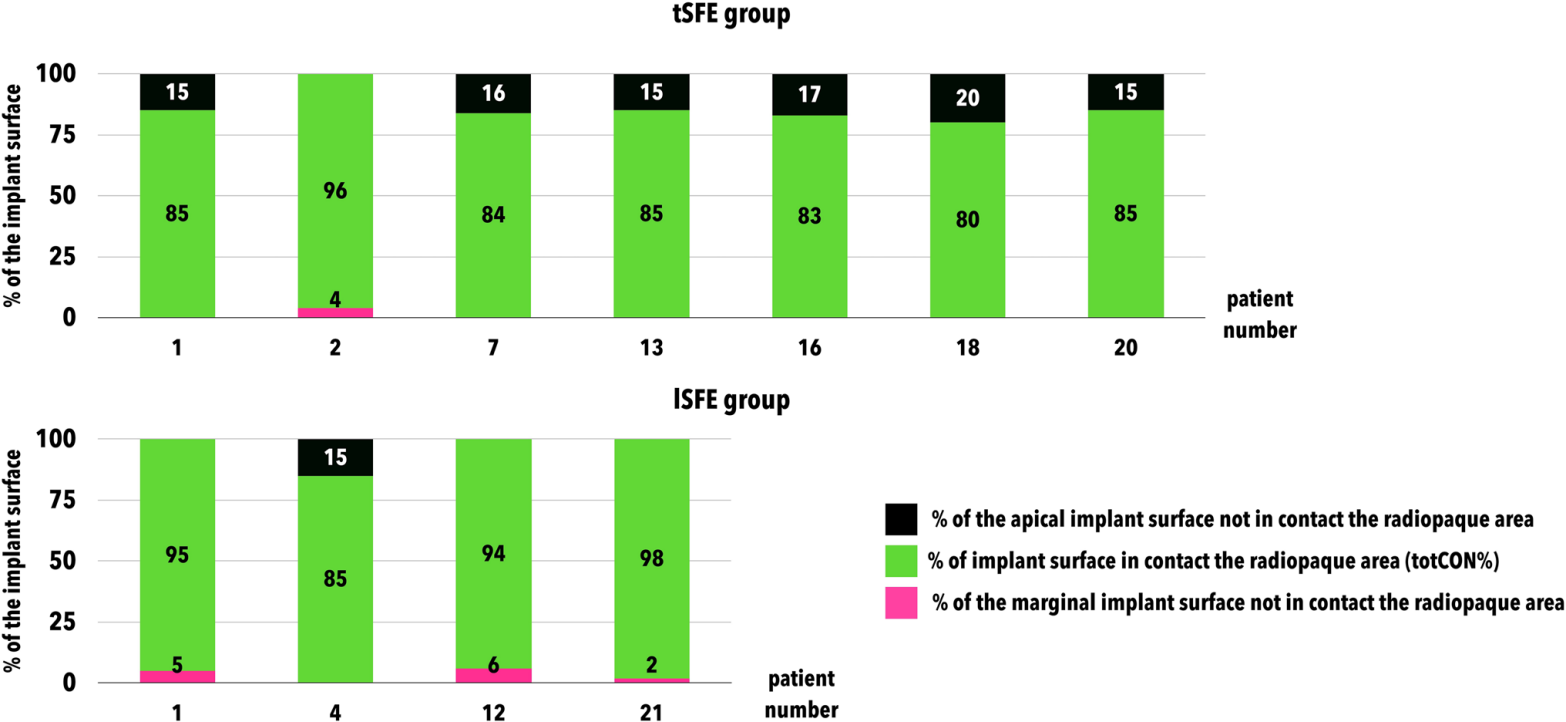
Exposure of the implant apex to either the maxillary sinus or oral cavity in cases with suboptimal (< 100%) totCON% values at 3 years in tSFE and lSFE groups

Raw data related to totCON% in tSFE and lSFE groups are reported in Fig. 3. At 1 year, median totCON% was 100% (IR: 100–100%) in both groups (*p* = 0.281). Mean 1-year totCON% values were 97.0% and 99.2% in tSFE and lSFE groups, respectively. The proportion of implants that showed increased, stable, or decreased totCON% values at 3 years compared to 1 year was 9.5%, 76.2%, and 14.3%, respectively, in the tSFE group, and 4.2%, 87.5%, and 8.3%, respectively, in the lSFE group. From 1-year to 3-year visit, within-group change in totCON% was not statistically significant in the tSFE group (*p* = 0.313). A limited 1–3-year variation in totCON% (which prevented inferential statistics) was observed in the lSFE group. At 3 years, median totCON% was 100% (IR: 84.6–100%) in the tSFE group and 100% (IR: 100–100%) in the lSFE group, with no significant inter-group difference (*p* = 0.124). Mean 3-year totCON% values were 95.1% and 98.8% in tSFE and lSFE groups, respectively. In the tSFE group, 14 patients (67%) had a 3-year totCON% of 100%, while 7 patients (33%) had lower totCON% values (80%, 83%, 84%, 85%, 85%, 85%, 96%). In the lSFE group, 20 patients (83%) had 3-year totCON% of 100%, while 4 patients (17%) had lower totCON% values (85%, 94%, 95%, 98%). The suboptimal (i.e., < 100%) 3-year totCON% values were mainly due to the exposure of the implant apex to the sinus cavity (Fig. 4).

### Secondary outcome measures

Raw data related to mMBL and dMBL in tSFE and lSFE groups are reported in Table 2. mMBL and dMBL values of 0 mm were observed in the vast majority of patients at both 1 and 3 years. In patients showing mMBL and/or dMBL apical to the implant shoulder at 1 year, a coronal migration of the marginal bone level was observed at 3 years. Only one case in lSFE showed a bone loss (0.30 mm) from 1 to 3 years. Median mMBL and dMBL was 0 (IR: 0–0) in both treatment groups at either 1 or 3 years, with no significant inter-group differences. A limited 1–3-year variation in mMBL and dMBL (which prevented inferential statistics) was observed in both groups.

**Table 2:** Raw data related to 1- and 3-year mMBL and dMBL in tSFE and lSFE groups. Negative changes in MBL indicate a coronal migration of the marginal bone level from 1-year to 3-year visit

Treatment group	Patient number	1-year mMBL (mm)	3-year mMBL (mm)	mMBL change (mm)	*p* value (intra-group comparison)	1-year dMBL (mm)	3-year dMBL (mm)	dMBL change (mm)	*p* value (intra-group comparison)
tSFE group (*n* = 21)	**1**	0	0	0		0	0	0	
	**2**	0.88	0.51	− 0.37		0.86	0.39	− 0.47	
	**3**	0	0	0		0	0	0	
	**4**	0.71	0	− 0.71		0	0	0	
	**5**	0	0	0		0	0	0	
	**6**	0	0	0		0	0	0	
	**7**	0.83	0	− 0.83		0	0	0	
	**8**	0	0	0		0	0	0	
	**9**	0	0	0		0	0	0	
	**10**	0	0	0		0	0	0	
	**11**	0	0	0		0	0	0	
	**12**	0	0	0		0	0	0	
	**13**	0	0	0		0	0	0	
	**14**	0	0	0		0	0	0	
	**15**	0	0	0		0	0	0	
	**16**	0	0	0		0	0	0	
	**17**	0	0	0		0	0	0	
	**18**	0	0	0		0	0	0	
	**19**	0	0	0		0	0	0	
	**20**	0	0	0		0	0	0	
	**21**	0	0	0		0	0	0	
Median		**0**	**0**	**0**	*	**0**	**0**	**0**	*
IR		**0–0**	**0–0**	**0–0**		**0–0**	**0–0**	**0–0**	
lSFE group (*n* = 24)	**1**	0	0.30	0.30		0.80	0.80	0	
	**2**	0	0	0		0	0	0	
	**3**	0	0	0		0	0	0	
	**4**	0	0	0		0	0	0	
	**5**	0	0	0		0	0	0	
	**6**	0	0	0		0	0	0	
	**7**	0	0	0		0	0	0	
	**8**	0	0	0		0	0	0	
	**9**	0	0	0		0	0	0	
	**10**	0	0	0		0	0	0	
	**11**	0	0	0		0	0	0	
	**12**	0.85	0.85	0		0.58	0.58	0	
	**13**	0	0	0		0	0	0	
	**14**	0	0	0		0	0	0	
	**15**	0	0	0		0	0	0	
	**16**	0	0	0		0	0	0	
	**17**	0	0	0		0	0	0	
	**18**	0	0	0		0	0	0	
	**19**	0	0	0		0	0	0	
	**20**	0	0	0		0	0	0	
	**21**	0	0	0		1.29	0.40	− 0.89	
	**22**	0	0	0		0	0	0	
	**23**	0	0	0		0	0	0	
	**24**	0	0	0		0	0	0	
Median		**0**	**0**	**0**	*	**0**	**0**	**0**	*
IR		**0–0**	**0–0**	**0–0**		**0–0**	**0–0**	**0–0**	
*p* value (inter-group comparison)		0.259	0.655	0.040		0.394	0.345	0.975	

^*^The limited variation in data between 1 and 3 years prevented the statistical comparison by the Wilcoxon test

Clinical measurements are reported in Table 3. Median PD was 3 mm, with most patients (tSFE: 95.2%; lSFE: 83.3%) showing PD ≤ 5 mm. Prevalence of BoP per patient was 16.7% in both groups, and all patients showed REC = 0 mm. No SoP + sites were recorded in both groups. No significant differences in any of the investigated clinical measurements were found between groups.

**Table 3:** Three-year clinical measurements in tSFE and lSFE groups

	tSFE group (*n* = 21)	lSFE group (*n* = 24)	*p* value
PD (mm)	3.0 (2.3–4.0)	3.0 (3.0–4.0)	0.217
Highest PD: 3 mm / 4 mm / 5 mm / 6 mm / 7 mm (no. of patients)	6 / 10 / 4 / 1 / 0	5 / 8 / 7 / 3 / 1	0.636
Proportion of peri-implant BoP + sites per patient (%)	16.7 (0–33.3)	16.7 (0–37.5)	0.290
Proportion of peri-implant SoP + sites per patient (%)	0 (0–0)	0 (0–0)	1
REC (mm)	0 (0–0)	0 (0–0)	1

### Conditions of the peri-implant tissues

Peri-implant health and mucositis were diagnosed in 10 (47.6%) and 11 (52.4%) subjects, respectively, in the tSFE group, and in 8 (33.3%) and 16 (66.7%) subjects, respectively, in the lSFE group. No significant inter-group difference in patient distribution according to peri-implant diagnosis was observed (*p* = 0.502). In subjects with peri-implant mucositis, the proportion of BoP-positive sites was 33% (IR: 25.0–33.3%) in the tSFE group and 33% (IR: 16.7–54.2%) in the lSFE group (*p* = 0.660).

## Discussion

As in previous analyses conducted on the present patient cohort [16] or other retrospective and prospective studies on sinus floor elevation procedures [8, 22], the proportion of the implant surface in direct contact with the radiopaque area was used to quantify the peri-implant bone support following sinus floor elevation. In our study, totCON% encompasses both portions of the implant which are in contact with (1) the pristine bone and (2) the endo-sinusal grafted space, which may become indistinguishable during the process of endo-sinusal bone formation and graft remodeling. At 3 years, the median totCON% was 100% in both groups, indicating that the peri‐implant bone support obtained at 1 year was fully maintained at 3 years for both tSFE and lSFE.

Some considerations may be advanced to explain the stability of peri-implant bone support overtime following sinus floor elevation. The high proportion of patients showing totCON% of 100% at 1 year may be partly ascribed to the considerable sinus floor elevation that can be obtained with DBBM in combination with tSFE [27, 28, 30] and lSFE [37, 38]. Also, a limited incidence of intra- and postoperative complications was observed in both groups [15]. In this respect, a sub-analysis conducted on the present cohort showed that the appropriate intraoperative management of membrane perforation resulted in a limited impact of membrane perforations on the 1-year radiographic outcomes, in general, and totCON%, in particular [16]. The stability in totCON% observed from 1 to 3 years may be partly explained by the use of DBBM that was shown to undergo slow resorption/degradation rate following sinus lift [39–42]. In particular, previous analyses showed that 72.6% of the vertical dimension of the area grafted with DBBM is maintained from 6 months to 3 years post-surgery [8]. Consistently with our results, a recent randomized trial reported that the use of DBBM resulted in similarly high and stable values of endo-sinus bone–implant contact rates (as assessed radiographically) for both tSFE and lSFE over a 2-year observation period [22].

Some technical aspects of the investigated interventions may have accounted for the minimal to null incidence and extent of MBL loss observed at 3 years post-surgery in both treatment groups. All patients received tissue-level implants with an intraoperatively conditioned hydrophilic surface. For this type of implants, stable levels of peri-implant marginal bone were reported at 3 [43] and 5 years [44] post-surgery, and similarly low apical migration of the peri-implant bone crest was reported at 2 years following their placement concomitantly with tSFE (0.35 mm) and lSFE (0.40 mm) in a randomized trial [14]. In the present material, implants were placed with the 1.0‐mm polished collar above the bone crest. Data from a prospective trial on the same implant system showed that the displacement of the implant-abutment junction at least 0.5 mm coronal to the bone crest had a positive impact on peri-implant bone loss at 1 year, which amounted to 0.2 mm and 0.4 mm for implants with 0.5-mm and 2.5-mm polished collar, respectively [45]. Interestingly, data from another randomized study where bone-level implants were used showed that the crestal bone level was always coronal to the implant shoulder in all tSFE cases and all but one lSFE cases at 2 years [22].

In the present study, the healing protocol (submerged or transmucosal) was left at the operator discretion. Due to different healing conditions and the need for additional surgery for implant uncovering in the submerged approach, it could be hypothesized that the healing protocol may have partly influenced our findings on MBL. A meta-analysis conducted on 11 clinical studies, however, reported a non-significant estimate of the difference in 12-month MBL of − 0.01 mm between submerged and transmucosal implant placement protocols [46]. Also, the same review indicated a limited impact of the healing protocol on the outcomes of bone augmentation procedures, although this conclusion was based on a limited amount of data [46].

At 3 years, peri-implant mucositis occurred in tSFE and lSFE groups with a similar prevalence (52.4% and 66.7%, respectively) and extent (33% BoP + sites per implant in both groups). Within the limitations due to the heterogeneity in case definitions of peri-implant mucositis that have been used through the years, our findings confirm that peri-implant mucositis is a highly prevalent biological complication at dental implants, including those placed concomitantly with sinus floor elevation procedures in the atrophic posterior maxilla. In this respect, subject-based summary estimates for the prevalence of peri-implant mucositis as reported in influential systematic reviews were 42.9% [47] and 46.83% [48]. Also, clinical trials conducted on implants placed after staged lSFE indicate that this complication is prevalent even at short-term (1 year) follow-up, reaching incidence higher than 60% [49]. The extent of peri-implant mucosal inflammation (expressed in terms of prevalence of BoP + sites per implant with mucositis) is also rather consistent with other studies, where implant-level mean BoP values amounted to 43% [50]. Among the factors that have been associated with BoP around dental implants [51, 52], some may explain the high prevalence of BoP in our cohort. Given the statistically significant positive relationship between BoP and PD [50, 51, 53, 54], the presence of sites with moderate PD in both treatment groups (Table 3) may have contributed the high BoP prevalence. Since BoP assessments were performed maintaining the implant-supported prosthesis in situ, some BoP-positive sites may also have been caused by improper use/angulation of the probe with consequent tissue trauma.

Despite the high prevalence of peri-implant mucositis, no peri-implantitis cases (or implant failures/loss due to peri-implantitis progression) were observed in either the tSFE or lSFE group. Some hypotheses can be advanced to explain the absence of peri-implantitis cases in the present study cohort. First, the follow-up (3 years) may have been too short for peri-implantitis to become clinically manifest. In this respect, the incidence of peri-implantitis was showed to increase at the increasing of the mean function time [47]. The exclusion of heavy smokers from the study during the screening phase and the low prevalence of current and former smokers in our treatment groups may have limited the negative effect of smoking on the risk for peri-implantitis [23, 55].

A potential limitation of the study may reside in the lack of data on 9 patients who participated in the 1-year visit, but were not available for the 3-year visit. Also, differently from a previous analysis [16], totCON% was assessed on bidimensional (periapical) radiographs taken without a customized film holder at both 1 and 3 years. Although periapical radiographs may suffer from dimensional distortion due to deformation of the film on the palate and allow for the evaluation of the mesial, distal, and apical implant aspect only, recent findings showed a high level of agreement between linear measurements of peri-implant bone anchorage performed on non-standardized periapical radiographs and CBCTs [56]. However, whether and to what extent a bidimensional evaluation of totCON% may reflect the peri-implant bone condition when assessed 360° around the implant by a CT/CBCT is currently undetermined, and the lack of a customized film holder remains a potential limitation of the present study that may have partly affected the reliability of within- and between-group comparisons. Another limitation is the lack of information on the amount of plaque deposits as well as the characteristics of supportive periodontal care from 1 to 3 years. In this respect, the onset and severity of peri-implant diseases has been previously associated with the amounts of plaque deposits [57, 58] as well as the efficacy of a supportive periodontal care regimen [59, 60].

## Conclusions

In conclusion, the results of the present study indicate that, at 3 years following surgery, implants placed concomitantly with tSFE and lSFE fully maintain peri-implant bone support. Peri-implant mucositis was the most prevalent condition, with a similar prevalence between groups.

## References

[1] Aludden H, Mordenfeld A, Hallman M, Christensen AE, Starch-Jensen T (2018). Osteotome-mediated sinus floor elevation with or without a grafting material: a systematic review and meta-analysis of long-term studies (≥5-years). Implant Dent.

[2] Raghoebar GM, Onclin P, Boven GC, Vissink A, Meijer HJA (2019). Long-term effectiveness of maxillary sinus floor augmentation: a systematic review and meta-analysis. J Clin Periodontol.

[3] Diserens V, Mericske E, Mericske-Stern R (2005). Radiographic analysis of the transcrestal sinus floor elevation: short-term observations. Clin Implant Dent Relat Res.

[4] Marković A, Mišić T, Calvo-Guirado JL, Delgado-Ruíz RA, Janjić B, Abboud M (2016). Two-center prospective, randomized, clinical, and radiographic study comparing osteotome sinus floor elevation with or without bone graft and simultaneous implant placement. Clin Implant Dent Relat Res.

[5] Nedir R, Nurdin N, Abi Najm S, El Hage M, Bischof M (2017). Short implants placed with or without grafting into atrophic sinuses: the 5-year results of a prospective randomized controlled study. Clin Oral Implants Res.

[6] Pjetursson BE, Ignjatovic D, Matuliene G, Brägger U, Schmidlin K, Lang NP (2009). Transalveolar maxillary sinus floor elevation using osteotomes with or without grafting material. Part II: radiographic tissue remodeling. Clin Oral Implants Res.

[7] Temmerman A, Van Dessel J, Cortellini S, Jacobs R, Teughels W, Quirynen M (2017). Volumetric changes of grafted volumes and the Schneiderian membrane after transcrestal and lateral sinus floor elevation procedures: a clinical, pilot study. J Clin Periodontol.

[8] Franceschetti G, Farina R, Minenna L, Riccardi O, Stacchi C, Di Raimondo R, Maietti E, Trombelli L (2020). The impact of graft remodeling on peri-implant bone support at implants placed concomitantly with transcrestal sinus floor elevation: a multicenter, retrospective case series. Clin Oral Implants Res.

[9] Coopman R, Fennis J, Ghaeminia H, Van de Vyvere G, Politis C, Hoppenreijs TJM (2020). Volumetric osseous changes in the completely edentulous maxilla after sinus grafting and lateral bone augmentation: a systematic review. Int J Oral Maxillofac Surg.

[10] Zitzmann NU, Schärer P (1998). Sinus elevation procedures in the resorbed posterior maxilla. Comparison of the crestal and lateral approaches. Oral Surg Oral Med Oral Pathol Oral Radiol Endod.

[11] Bensaha T (2011). Evaluation of the capability of a new water lift system to reduce the risk of Schneiderian membrane perforation during sinus elevation. Int J Oral Maxillofac Surg.

[12] Kim SM, Park JW, Suh JY, Sohn DS, Lee JM (2011). Bone-added osteotome technique versus lateral approach for sinus floor elevation: a comparative radiographic study. Implant Dent.

[13] Al-Almaie S, Kavarodi AM, Alorf A, Alzahrani S (2017). A split-mouth design comparison for lateral and crestal sinus lift techniques with dental implants placements: short communication. Open Dent J.

[14] Yu H, Wang X, Qiu L (2017). Outcomes of 6.5-mm hydrophilic implants and long implants placed with lateral sinus floor elevation in the atrophic posterior maxilla: a prospective, randomized controlled clinical comparison. Clin Implant Dent Relat Res.

[15] Farina R, Franceschetti G, Travaglini D, Consolo U, Minenna L, Schincaglia GP, Riccardi O, Bandieri A, Maietti E, Trombelli L (2018). Morbidity following transcrestal and lateral sinus floor elevation: a randomized trial. J Clin Periodontol.

[16] Farina R, Franceschetti G, Travaglini D, Consolo U, Minenna L, Schincaglia GP, Riccardi O, Bandieri A, Maietti E, Trombelli L (2019). Radiographic outcomes of transcrestal and lateral sinus floor elevation: one-year results of a bi-center, parallel-arm randomized trial. Clin Oral Implants Res.

[17] Farina R, Simonelli A, Franceschetti G, Travaglini D, Consolo U, Minenna L, Schincaglia GP, Riccardi O, Bandieri A, Trombelli L (2021). Implant-supported rehabilitation following transcrestal and lateral sinus floor elevation: analysis of costs and quality of life from a bi-center, parallel-arm randomized trial. Minerva Dent Oral Sci. 2021 (in press, epub ahead of print). 10.23736/S2724-6329.21.04539-3 33988332

[18] Cannizzaro G, Felice P, Leone M, Viola P, Esposito M (2009). Early loading of implants in the atrophic posterior maxilla: lateral sinus lift with autogenous bone and Bio-Oss versus crestal mini sinus lift and 8-mm hydroxyapatite-coated implants. A randomised controlled clinical trial. Eur J Oral Implantol.

[19] Cannizzaro G, Felice P, Minciarelli AF, Leone M, Viola P, Esposito M (2013). Early implant loading in the atrophic posterior maxilla: 1-stage lateral versus crestal sinus lift and 8 mm hydroxyapatite-coated implants. A 5-year randomized controlled trial. Eur J Oral Implantol.

[20] Tsai CF, Pan WL, Pan YP, Chan CP, Ju YR, Wang YM, Lin CY, Chang CC (2020). Comparison of 4 sinus augmentation techniques for implant placement with residual alveolar bone height ≤3 mm. Medicine (Baltimore) 99:e23180. 10.1097/MD.0000000000023180 PMC7668509 33181695

[21] Bacevic M, Compeyron Y, Lecloux G, Rompen E, Lambert F (2021). Intraoperative and postoperative outcomes of sinus floor elevation using the lateral window technique versus the hydrodynamic transalveolar approach: a preliminary randomized controlled trial. Clin Oral Investig.

[22] Zhou Y, Shi Y, Si M, Wu M, Xie Z (2021). The comparative evaluation of transcrestal and lateral sinus floor elevation in sites with residual bone height ≤6 mm: a two-year prospective randomized study. Clin Oral Implants Res.

[23] Stacchi C, Troiano G, Rapani A, Lombardi T, Sentineri R, Speroni S, Berton F, Di Lenarda R (2021). Factors influencing the prevalence of peri-implantitis in implants inserted in augmented maxillary sinuses: a multicenter cross-sectional study. J Periodontol.

[24] Trombelli L, Minenna P, Franceschetti G, Farina R, Minenna L (2008). Smart Lift: una nuova procedura minimamente invasiva per la elevazione del pavimento del seno mascellare. Dental Cadmos 76:71–83. (article in italian).

[25] Trombelli L, Minenna P, Franceschetti G, Minenna L, Itro A, Farina R (2010). Minimally invasive technique for transcrestal sinus floor elevation: a case report. Quintessence Int.

[26] Trombelli L, Minenna P, Franceschetti G, Minenna L, Farina R (2010). Transcrestal sinus floor elevation with a minimally invasive technique. J Periodontol.

[27] Trombelli L, Franceschetti G, Rizzi A, Minenna P, Minenna L, Farina R (2012). Minimally invasive transcrestal sinus floor elevation with graft biomaterials. A randomized clinical trial. Clin Oral Implants Res.

[28] Trombelli L, Franceschetti G, Stacchi C, Minenna L, Riccardi O, Di Raimondo R, Rizzi A, Farina R (2014). Minimally invasive transcrestal sinus floor elevation with deproteinized bovine bone or β-tricalcium phosphate: a multicenter, double-blind, randomized, controlled clinical trial. J Clin Periodontol.

[29] Trombelli L, Franceschetti G, Trisi P, Farina R (2015). Incremental, transcrestal sinus floor elevation with a minimally invasive technique in the rehabilitation of severe maxillary atrophy. Clinical and histological findings from a proof-of-concept case series. J Oral Maxillofac Surg.

[30] Franceschetti G, Farina R, Stacchi C, Di Lenarda R, Di Raimondo R, Trombelli L (2014). Radiographic outcomes of transcrestal sinus floor elevation performed with a minimally invasive technique in smoker and non-smoker patients. Clin Oral Implants Res.

[31] Franceschetti G, Trombelli L, Minenna L, Franceschetti G, Farina R (2015). Learning curve of a minimally invasive technique for transcrestal sinus floor elevation: a split-group analysis in a prospective case series with multiple clinicians. Implant Dent.

[32] Franceschetti G, Rizzi A, Minenna L, Pramstraller M, Trombelli L, Farina R (2017). Patient-reported outcomes of implant placement performed concomitantly with transcrestal sinus floor elevation or entirely in native bone. Clin Oral Implants Res.

[33] Trombelli L, Farina R, Ferrari S, Pasetti P, Calura G (2009). Comparison between two methods for periodontal risk assessment. Minerva Stomatol.

[34] Trombelli L, Minenna L, Toselli L, Zaetta A, Checchi L, Checchi V, Nieri M, Farina R (2017). Prognostic value of a simplified method for periodontal risk assessment during supportive periodontal therapy. J Clin Periodontol.

[35] Farina R, Simonelli A, Baraldi A, Pramstraller M, Minenna L, Toselli L, Maietti E, Trombelli L (2021). Tooth loss in complying and non-complying periodontitis patients with different periodontal risk levels during supportive periodontal care. Clin Oral Investig.

[36] Berglundh T, Armitage G, Araujo MG, Avila-Ortiz G, Blanco J, Camargo PM, Chen S, Cochran D, Derks J, Figuero E, Hämmerle CHF, Heitz-Mayfield LJA, Huynh-Ba G, Iacono V, Koo KT, Lambert F, McCauley L, Quirynen M, Renvert S, Salvi GE, Schwarz F, Tarnow D, Tomasi C, Wang HL, Zitzmann N (2018). Peri-implant diseases and conditions: consensus report of workgroup 4 of the 2017 World Workshop on the Classification of Periodontal and Peri-Implant Diseases and Conditions. J Periodontol.

[37] Chackartchi T, Iezzi G, Goldstein M, Klinger A, Soskolne A, Piattelli A, Shapira L (2011). Sinus floor augmentation using large (1–2 mm) or small (0.25-1 mm) bovine bone mineral particles: a prospective, intra-individual controlled clinical, micro-computerized tomography and histomorphometric study. Clin Oral Implants Res.

[38] Merli M, Moscatelli M, Mariotti G, Rotundo R, Nieri M (2013). Autogenous bone versus deproteinised bovine bone matrix in 1-stage lateral sinus floor elevation in the severely atrophied maxilla: a randomised controlled trial. Eur J Oral Implantol.

[39] Lee YM, Shin SY, Kim JY, Kye SB, Ku Y, Rhyu IC (2006). Bone reaction to bovine hydroxyapatite for maxillary sinus floor augmentation: histologic results in humans. Int J Periodontics Restorative Dent.

[40] Traini T, Valentini P, Iezzi G, Piattelli A (2007). A histologic and histomorphometric evaluation of anorganic bovine bone retrieved 9 years after a sinus augmentation procedure. J Periodontol.

[41] Mordenfeld A, Hallman M, Johansson CB, Albrektsson T (2010). Histological and histomorphometrical analyses of biopsies harvested 11 years after maxillary sinus floor augmentation with deproteinized bovine and autogenous bone. Clin Oral Implants Res.

[42] Pettinicchio M, Traini T, Murmura G, Caputi S, Degidi M, Mangano C, Piattelli A (2012). Histologic and histomorphometric results of three bone graft substitutes after sinus augmentation in humans. Clin Oral Investig.

[43] Hicklin SP, Janner SF, Schnider N, Chappuis V, Buser D, Brägger U (2020). Early loading of titanium dental implants with an intraoperatively conditioned hydrophilic implant surface: 3-year results of a prospective case series study. Int J Oral Maxillofac Implants.

[44] Gholami H, Mericske-Stern R, Kessler-Liechti G, Katsoulis J (2014). Radiographic bone level changes of implant-supported restorations in edentulous and partially dentate patients: 5-year results. Int J Oral Maxillofac Implants.

[45] van Eekeren PJ, Tahmaseb A, Wismeijer D (2016). Crestal bone changes around implants with implant-abutment connections at epicrestal level or above: systematic review and meta-analysis. Int J Oral Maxillofac Implants.

[46] Paul S, Petsch M, Held U (2017). Modeling of crestal bone after submerged vs transmucosal implant placement: a systematic review with meta-analysis. Int J Oral Maxillofac Implants.

[47] Derks J, Tomasi C (2015). Peri-implant health and disease. A systematic review of current epidemiology. J Clin Periodontol 42 Suppl 16:S158–171. 10.1111/jcpe.12334 25495683

[48] Lee CT, Huang YW, Zhu L, Weltman R (2017). Prevalences of peri-implantitis and peri-implant mucositis: systematic review and meta-analysis. J Dent.

[49] Alayan J, Ivanovski S (2019). Biological and technical outcomes of restored implants after maxillary sinus augmentation—results at 1-year loading. Clin Oral Implants Res.

[50] Ramanauskaite A, Becker K, Schwarz F (2018). Clinical characteristics of peri-implant mucositis and peri-implantitis. Clin Oral Implants Res.

[51] Farina R, Filippi M, Brazzioli J, Tomasi C, Trombelli L (2017). Bleeding on probing around dental implants: a retrospective study of associated factors. J Clin Periodontol.

[52] Monje A, Nart J, Amerio E, Farina R, Trombelli L, Roccuzzo A, Salvi GE, Schwarz F, Ramanauskaite A, Renvert S, Wang H-L (2021). Significance of probing for monitoring peri-implant conditions. Int J Oral Implantol (Berl).

[53] Seki K, Nakabayashi S, Tanabe N, Kamimoto A, Hagiwara Y (2017). Correlations between clinical parameters in implant maintenance patients: analysis among healthy and history-of-periodontitis groups. Int J Implant Dent.

[54] Merli M, Bernardelli F, Giulianelli E, Toselli I, Mariotti G, Nieri M (2017). Peri-implant bleeding on probing: a cross-sectional multilevel analysis of associated factors. Clin Oral Implants Res.

[55] Atieh MA, Alsabeeha NH, Faggion CM, Duncan WJ (2013). The frequency of peri-implant diseases: a systematic review and meta-analysis. J Periodontol.

[56] El Hage M, Nurdin N, Abi Najm S, Bischof M, Nedir R (2019). Osteotome sinus floor elevation without grafting: a 10-year study of cone beam computerized tomography vs periapical radiography. Int J Periodontics Restorative Dent.

[57] Salvi GE, Aglietta M, Eick S, Sculean A, Lang NP, Ramseier CA (2012). Reversibility of experimental peri-implant mucositis compared with experimental gingivitis in humans. Clin Oral Implants Res.

[58] Romandini M, Lima C, Pedrinaci I, Araoz A, Soldini MC, Sanz M (2021). Prevalence and risk/protective indicators of peri-implant diseases: a university-representative cross-sectional study. Clin Oral Implants Res.

[59] Monje A, Aranda L, Diaz KT, Alarcón MA, Bagramian RA, Wang HL, Catena A (2016). Impact of maintenance therapy for the prevention of peri-implant diseases: a systematic review and meta-analysis. J Dent Res.

[60] Ramanauskaite A, Tervonen T (2016). The efficacy of supportive peri-implant therapies in preventing peri-implantitis and implant loss: a systematic review of the literature. J Oral Maxillofac Res 7:e12. 10.5037/jomr.2016.7312 PMC5100637 27833737

